# Cu/Fe co-doped *Sphagnum palustre*-derived biochar for the synergistic adsorption and photocatalytic removal of tetracycline hydrochloride

**DOI:** 10.1016/j.jare.2025.12.051

**Published:** 2025-12-29

**Authors:** Qing Xiang, Zhen Wang, Liang Luo, Yu Fang, Yuheng Cui, Junbo Zhou, Daixiong Zhang, Bo Yang, Zhaohui Zhang, Xuefeng Zou, Bin Xiang

**Affiliations:** a School of Materials and Architectural Engineering, Guizhou Normal University, Guiyang 550001, PR China; b College of Chemistry and Chemical Engineering, Chongqing University, Chongqing 401331, PR China; c Faculty of Architecture, Building and Planning, The University of Melbourne, VIC 3052, Australia; d College of Environment and Resources, Chongqing Key Laboratory of Catalysis and New Environmental Materials, Chongqing Technology and Business University, Chongqing 400067, PR China; e Information System and Protection of Key Laboratory in Mountainous Areas of Guizhou Normal University, Guizhou Normal University, Guiyang 550001, PR China; f Key Laboratory of Guizhou Institute of Computer Nanomaterials Science, Guizhou Education University, Guiyang 550018, PR China

**Keywords:** *Sphagnum palustre*, Cu/Fe co-doped biochar, Visible-light photocatalysis, Adsorption-photocatalysis synergy, Tetracycline hydrochloride removal

## Abstract

**Introduction:**

The widespread presence of antibiotic pollutants, such as tetracycline hydrochloride (TCH), causes significant environmental and public health concerns. Biochar-based photocatalysts derived from renewable biomass have attracted increasing attention due to their low cost, structural tunability, and environmental sustainability. However, their photocatalytic performance is often limited by poor charge separation and a lack of active sites.

**Objectives:**

This study aims to construct a visible-light-responsive Cu/Fe co-doped biochar composite using *Sphagnum palustre* as a biomass precursor for the synergistic adsorption and photocatalytic removal of TCH from aqueous environments.

**Methods:**

The Cu/Fe co-doped photocatalyst (CFO/S) was synthesized via a hydrothermal method by integrating Cu–Fe oxides with *Sphagnum*-derived biochar. The composite was comprehensively characterized, and its visible-light performance was evaluated. The photocatalytic mechanism was elucidated through radical trapping experiments and DFT+U simulations.

**Results:**

The CFO/S-10 composite achieved a TCH removal efficiency of 94.56% within 60 min under visible-light irradiation. Adsorption was identified as the primary removal mechanism, while photocatalysis contributed to the degradation of adsorbed molecules. A layered FeO/CuFe_2_O_4_/S structure promoted charge separation and intermediate desorption. Multiple degradation products were detected, involving demethylation, hydroxylation, and ring-opening reactions.

**Conclusion:**

The Cu/Fe co-doped biochar composite exhibited excellent removal performance through a synergistic adsorption-photocatalysis mechanism. Photogenerated electrons were the dominant reactive species, supported by •OH, •O_2_^−^, and h^+^. An S-scheme charge transfer mechanism was proposed to explain the enhanced redox capability. These findings demonstrate the potential of CFO/S as a promising candidate for visible-light-driven removal of antibiotic contaminants in water.

## Highlights

• Sustainable Cu–Fe oxide photocatalyst synthesized from S. palustre biochar.
• Cu–Fe co‑doping enhanced charge transfer and intermediate desorption.
• CFO/S‑10 removed 94.6 % of TCH via adsorption–photocatalysis synergy.
• Heterojunction promoted charge separation and ROS generation.

## Graphical abstract

A Cu/Fe co-doped *S. palustre*-derived biochar composite with an S-scheme heterojunction enables synergistic adsorption and photocatalytic removal of tetracycline hydrochloride under visible-light irradiation, achieving an overall efficiency of 94.56%.

**Figure:**
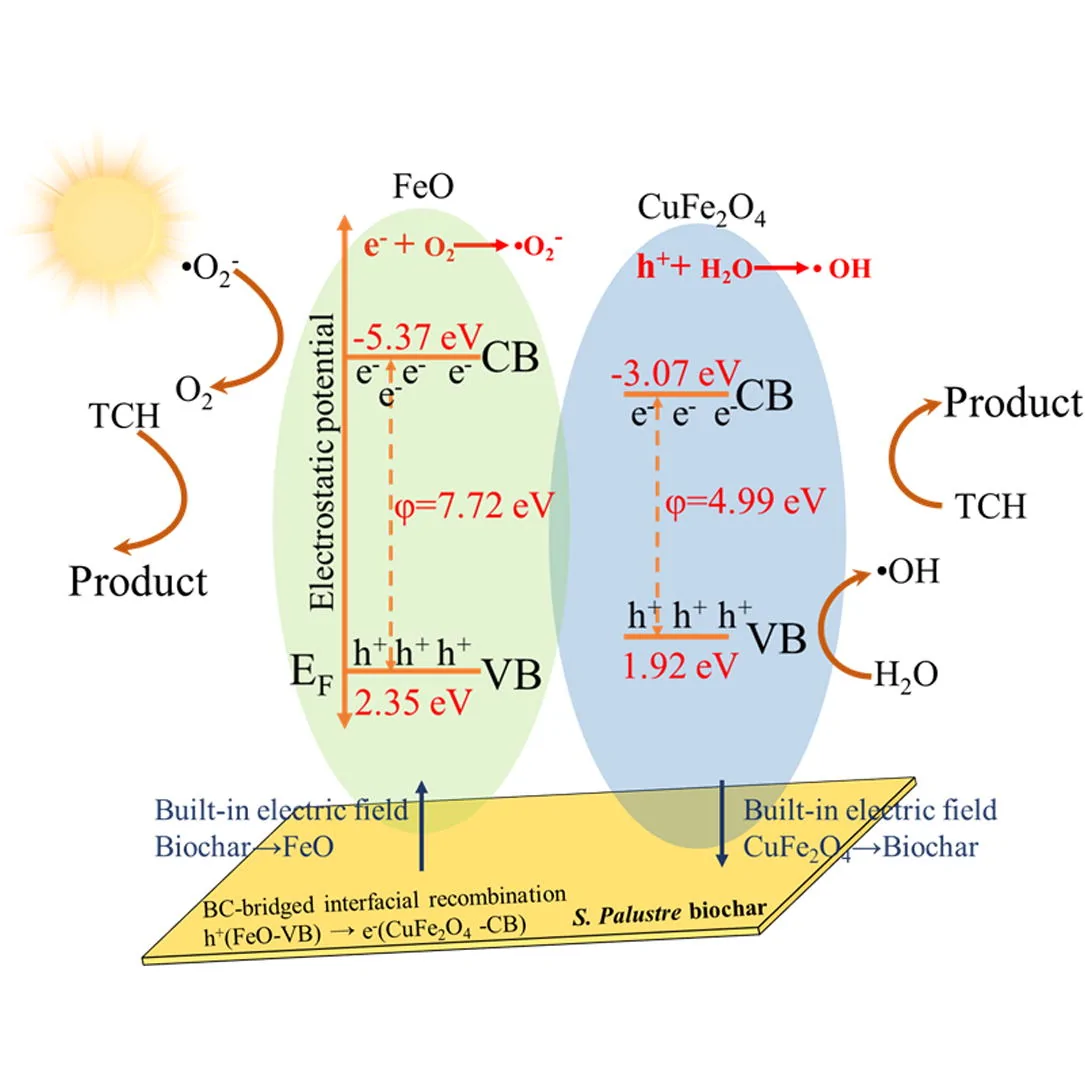

## Introduction

The widespread use and continuous release of antibiotics such as tetracycline hydrochloride (TCH), ofloxacin, and other ionizable pharmaceutical compounds have led to their persistent accumulation in aquatic environments [1], [2]. These emerging contaminants exhibit bioaccumulative toxicity, resistance to natural biodegradation, and facilitate the spread of antibiotic resistance genes (ARGs), thereby posing serious threats to both environmental and public health [3]. Among them, TCH is particularly persistent due to its molecular stability and strong sediment adsorption, making its effective removal from wastewater a significant environmental challenge [4].

Visible-light-driven photocatalysis offers a promising solution owing to its mild conditions, low energy consumption, and ability to mineralize organic pollutants into harmless products [5], [6]. In this process, semiconductor photocatalysts generate electron-hole (e^−^/h^+^) pairs under light irradiation, which then produce reactive oxygen species (ROS), such as hydroxyl radicals (•OH) and superoxide radicals (•O_2_^−^), responsible for degrading complex organic molecules [7], [8]. However, practical application of photocatalysis is limited by rapid charge recombination, narrow visible-light response, and poor long-term stability.

Constructing heterojunction photocatalysts can address these limitations by enhancing charge separation through interfacial polarization and internal electric fields, thereby improving photocatalytic performance under visible light [9], [10]. Moreover, combining semiconductors with conductive carbonaceous materials can further facilitate interfacial charge transfer and suppress recombination.

Biochar, a porous carbonaceous material derived from biomass pyrolysis, has gained attention as a sustainable support for photocatalysts [11]. Its high specific surface area, abundant oxygen-containing functional groups (e.g., –OH and –COOH), and π–π interactions enable efficient adsorption of aromatic antibiotics like TCH, and promote charge transfer during photocatalysis [12]. Importantly, the physicochemical properties of biochar depend heavily on the biomass precursor. *Sphagnum palustre* L. (*S. palustre*), a naturally abundant wetland moss rich in carbonaceous components and oxygen-containing functional groups, yields biochar with high porosity, strong interfacial activity, and excellent stability, making it ideal for advanced photocatalyst fabrication [13], [14].

Functional modification of biochar with transition metals can further enhance photocatalytic activity. In particular, co-doping with copper (Cu) and iron (Fe) offers synergistic benefits over single-metal incorporation, as their complementary redox pairs (Cu^+^/Cu^2+^ and Fe^2+^/Fe^3+^) support continuous ROS generation under visible light [15], [16]. Compared with other transition metals, Cu and Fe are more abundant, cost-effective, and environmentally compatible. Their uniform incorporation into the biochar matrix promotes the formation of stable heterojunction interfaces, facilitating charge separation and reducing metal leaching risks, which is critical for environmental safety and long-term catalytic performance [17].

Despite these advantages, designing Cu–Fe/biochar-based heterojunctions with optimized charge transfer pathways and interfacial structure remains challenging. In this study, a Cu/Fe co-doped heterojunction photocatalyst (denoted CFO/S) was synthesized via a one-pot hydrothermal method using *S. palustre*-derived biochar as the structural platform. The resulting material exhibited a hierarchically porous architecture with uniformly distributed Cu–Fe species, forming an integrated heterojunction interface that facilitated charge separation and improved electron transport under visible-light irradiation. The CFO/S catalyst achieved 94.56% removal of TCH within 60 min. Comprehensive physicochemical characterization combined with spin-polarized DFT+U simulations revealed that the asymmetric charge distribution induced an internal electric field, which significantly promoted charge separation and ROS generation.

This work provides a green and efficient strategy for valorizing underutilized wetland biomass and offers new insights into interfacial charge modulation in Cu–Fe/biochar heterojunctions, contributing to the rational design of high-performance photocatalysts for removing emerging contaminants from aquatic environments.

## Experimental

### Chemical reagents

All chemical reagents were of analytical grade and used without further purification. *S. palustre* was collected from Gaopo Township, Guiyang, Guizhou Province, China. The reagents included sodium carboxymethyl cellulose (CMC-Na), ferric chloride hexahydrate (FeCl_3_·6H_2_O), copper chloride dihydrate (CuCl_2_·2H_2_O), tetracycline hydrochloride (TCH, C_22_H_24_N_2_O_8_), levofloxacin (LVX, C_18_H_20_FN_3_O_4_), *tert*-butanol (t-BuOH), disodium ethylenediaminetetraacetate (EDTA-2Na), benzoquinone (BQ), and potassium dichromate (K_2_Cr_2_O_7_). All chemicals were supplied by Macklin (China).

### Sample preparation

*S. palustre* was thoroughly washed with deionized water, vacuum-dried at 60 °C, and ground into a fine powder. The composite catalyst CFO/S was synthesized via a hydrothermal method.

In a typical synthesis, 0.30 g of CMC-Na (used as a dispersant and soft template) and 200 mg of *S. palustre* powder were dispersed in 50 mL of deionized water and stirred for 30 min to obtain a uniform viscous suspension. Then, 325 mg of FeCl_3_·6H_2_O and 111 mg of CuCl_2_·2H_2_O were added and stirred for 1 h to allow complete adsorption of metal ions onto the biochar precursor.

The solution pH was adjusted to 10 via a two-step titration. Initially, a 0.1 mol L^−1^ NaOH solution was added dropwise while monitoring the pH, raising it to approximately 9.0. Subsequently, a 0.01 mol L^−1^ NaOH solution was used for fine adjustment to precisely reach pH 10. This controlled approach ensured accurate alkalinity without localized over-alkalization. The mixture was further stirred for 2 h to ensure full complexation and homogeneous ion distribution. To ensure that the observed variations among the CFO/S samples arose solely from hydrothermal kinetics, the Cu:Fe molar ratio and total metal precursor dosage were deliberately kept constant across all syntheses. This experimental control ensured that differences in crystallinity, phase evolution, and interfacial properties could be primarily attributed to reaction time rather than compositional fluctuations.

The resulting suspension was transferred into a 100 mL Teflon-lined stainless steel autoclave and subjected to hydrothermal treatment at 150 ± 1 °C for 5, 10, 15, 20, or 24 h. The filling ratio was maintained at 50% to ensure adequate pressure buildup and efficient heat transfer while preventing overpressure. After the reaction, the system was allowed to cool naturally. The resulting product was washed repeatedly with deionized water and ethanol by centrifugation until the pH of the supernatant was neutral. The solid was then dried overnight at 60 °C under vacuum. The samples obtained after different hydrothermal durations were labeled as CFO/S-5, CFO/S-10, CFO/S-15, CFO/S-20, and CFO/S-24, respectively. Sample prepared without *S. palustre* was denoted as CFO, and the biochar without metal loading was referred to as S.

All syntheses were repeated at least three times under identical conditions to ensure reproducibility. The resulting materials showed consistent morphology and photocatalytic behavior across batches. Hydrothermal synthesis was employed due to its effectiveness in facilitating heterojunction formation, although it is recognized as a relatively energy-intensive method [18].

### Characterization

#### Morphology characterization.

Surface morphology and microstructure of the samples were examined using scanning electron microscopy (SEM, ZEISS Sigma 300) and transmission electron microscopy (TEM, JEOL JEM 2100F and FEI Talos F200X G2, 200 kV). For TEM imaging, samples were ultrasonically dispersed in ethanol and deposited onto carbon-coated copper grids by drop-casting. Energy-dispersive X-ray spectroscopy (EDX) and elemental mapping were conducted using an Oxford X-Max 80 T detector.

#### Phase structure analysis

X-ray diffraction (XRD, Bruker D8 Advance Eco) was employed to investigate the crystalline structure using Cu Kα radiation (λ = 1.5406 Å) at 40 kV and 40 mA. Diffraction patterns were recorded over a 2θ range of 10–90° at a scan rate of 5°/min.

X-ray absorption spectroscopy (XAS), including XANES and EXAFS, was carried out at the BL14W1 beamline of the Shanghai Synchrotron Radiation Facility (SSRF) to examine the oxidation states and coordination environments of Cu and Fe. The storage ring operated at 8.0 GeV with a beam current of 99.5 mA. Spectra were collected in transmission mode, and the data were processed using the Demeter software package. Parameters such as coordination number, bond distance, and Debye–Waller factor were extracted from EXAFS fitting results and summarized in Table 1, Table 2. The results suggested the presence of Cu–O, Cu–Cu, Fe–O, and Fe–Fe coordination, indicative of mixed-valence states in CFO/S-10.

**Table 1:** EXAFS fitting parameters at the Fe K-edge for the COF/S-10 samples (*S*_0_^2^ = 0.85).

	shell	CN^a^	R^b^ (Å)	σ^2^^c^ (Å^2^)	ΔE_0_^d^ (eV)	R factor
Fe-foil	Fe-Fe1	8	2.47±0.01	0.0052	6.0±1.6	0.0036
	Fe- Fe2	6	2.85±0.01	0.0062		

Fe0.68	Fe-O	4.74±0.3	1.93±0.02	0.0091	−7.0±1.7	0.01013
	Fe-Fe1	1.59±0.5	2.95±0.06	0.0097		
	Fe-Fe2	6.11±0.6	3.43±0.06	0.0098		

a *CN*: coordination numbers.

b *R*: bond distance.

c *σ*^2^: Debye-Waller factors.

d Δ*E*_0_: the inner potential correction. R factor: goodness of fit. Error bounds that characterize the structural parameters obtained by EXAFS spectroscopy were estimated as CN±20%; R ± 1%; σ^2^ ±20%.

**Table 2:** EXAFS fitting parameters at the Cu K-edge for the COF/S-10 samples(*S*_0_^2^ = 0.75 from Cu-foil).

	shell	CN^a^	R^b^ (Å)	σ^2^^c^ (Å^2^)	ΔE_0_^d^ (eV)	R factor
Cu-foil	Cu-Cu	12	2.54 ± 0.01	0.0086	4.6 ± 0.5	0.0023
Cu-Sample	Cu–O	4.56 ± 0.48	1.85 ± 0.02	0.0063	3.6 ± 1.7	0.0195
	Cu-Cu	5.72 ± 0.32	2.53 ± 0.02	0.0076		

a *CN*: coordination numbers.

b *R*: bond distance.

c *σ*^2^: Debye-Waller factors.

d Δ*E*_0_: the inner potential correction. R factor: goodness of fit. Error bounds that characterize the structural parameters obtained by EXAFS spectroscopy were estimated as CN ± 20 %; R ± 1 %; σ^2^ ± 20 %.

#### Surface chemistry characterization

Fourier-transform infrared spectroscopy (FTIR) was performed using a Bruker Tensor 27 spectrometer (Bruker Optik GmbH, Germany). X-ray photoelectron spectroscopy (XPS, ULVAC-PHI 5000) was employed to analyze surface composition and chemical states. Measurements were carried out under ultra-high vacuum (below 2.0 × 10^−7^ mbar) using a monochromatic Al Kα source (1486.6 eV) with a spot size of 400 μm. Survey spectra were acquired at 150 eV pass energy, and high-resolution spectra at 50 eV.

#### Optical properties, charge carrier dynamics and reactive species analysis

The fluorescence spectra of the samples were measured using a Hitachi F-7000 fluorescence spectrophotometer at an excitation wavelength of 330 nm to investigate the correlation properties of the electronic structures. Transient photoluminescence (PL) decay curves were recorded on an FS5 fluorescence lifetime spectrophotometer (Edinburgh Instruments, UK) with an excitation wavelength of 325 nm.

UV–visible diffuse reflectance spectroscopy (DRS) was performed on a U4100 ultraviolet spectrometer (Hitachi, Japan) with an excitation wavelength of 300 nm, a response voltage of 500 V, and a scanning wavelength range of 200–800 nm.

Electron Paramagnetic Resonance (EPR) spectroscopy was performed using a Bruker A300 spectrometer to probe photogenerated charge carriers generated during the photocatalytic process. TEMPO (20 mM) was employed as a spin-trapping agent to capture photogenerated electrons (e^−^) and holes (h^+^). EPR spectra were recorded after visible-light irradiation for 0, 4, and 8 min.

### Adsorption-photocatalytic experiment

Photocatalytic degradation tests were performed in a 250 mL degradation reactor. 5 mL of a 1 g/L TCH solution was diluted to 100 mL with deionized water and then filtered through a 0.22 μm filter. The initial absorbance was recorded at λ_max_ = 356 nm. Subsequently, 20 mg of CFO/S catalyst was added, and the suspension was stirred in the dark for 30 min to achieve adsorption–desorption equilibrium.

Visible-light irradiation was supplied by a 300 W xenon lamp (CEL-HXF300) equipped with a 420 nm cutoff filter, providing an intensity of approximately 100 mW/cm^2^ at the solution surface. During the reaction, 5 mL aliquots were withdrawn at 10-minute intervals, filtered, and analyzed by UV-vis spectrophotometry. Each aliquot was discarded after measurement to avoid cross-contamination.

In addition, high-performance liquid chromatography (HPLC, Scion LC6000) was employed to quantitatively monitor the concentration of TCH. Chromatographic separation was carried out on a C18 column using a mobile phase consisting of acetonitrile and 0.025 mol/L disodium hydrogen phosphate buffer, with a flow rate of 1.0 mL/min and a column temperature of 25 °C. The injection volume was 20 µL, and the retention time of TCH was approximately 4.6 min. In some low-concentration samples, a trace amount of EDTA-2Na was added to the buffer solution to improve peak stability, while blank and high-concentration samples were analyzed without EDTA. Quantification was based on peak area integration.

The degradation efficiency (DE, %) was calculated as:

$$\mathrm{DE}\left(\%\right)=\left(A_{0}-A_{t}\right)/A_{0}\times 100\%$$

where A_0_ and A_t_ are the absorbance values before and after irradiation, respectively.

The influence of *S. palustre* content (0–0.25 g L^−1^), solution pH (8–12), reaction temperature (25–45 °C), and light intensity (70–110 mW/cm^2^) on the photocatalytic removal efficiency of TCH was systematically evaluated.

To gain insights into the degradation mechanism, reactive species trapping experiments were performed using K_2_Cr_2_O_7_ (electron scavenger), CH_3_OH (general radical scavenger), t-BuOH (•OH scavenger), and EDTA-2Na (hole scavenger). These tests aimed to identify the predominant ROS involved in the photocatalytic process.

The recyclability and stability of the CFO/S-10 catalyst were assessed over five consecutive photocatalytic cycles under optimized conditions. After each cycle, the catalyst was recovered, washed, dried, and reused without significant loss of activity, demonstrating good operational stability.

The photocatalytic activity of CFO/S-10 was further evaluated against levofloxacin (LVX, C_18_H_20_FN_3_O_4_) as a representative antibiotic. Experiments were conducted under the same conditions as for TCH, except that LVX concentration was monitored at λ_max_ = 295 nm.

Degradation intermediates were analyzed by high-performance liquid chromatography coupled with time-of-flight mass spectrometry (HPLC–TOF-MS, Agilent 1290). Chromatographic separation was performed on a C18 column (1.7 µm) using a mobile phase of methanol and 0.1% formic acid at 0.3 mL/min flow rate. The injection volume was 5 µL.

### Simulation calculations

To elucidate the photocatalytic mechanism at the atomic level, spin-polarized density functional theory (DFT) + U calculations were conducted using the DS-PAW module in the HZWTECH package. The generalized gradient approximation (GGA) with the Perdew–Burke–Ernzerhof (PBE) exchange–correlation functional was employed. The core-valence interaction was described by the projected augmented wave (PAW) method with a kinetic energy cutoff of 500 eV.

The Brillouin zone was sampled with a 2 × 2 × 1 Monkhorst–Pack k-point mesh. The convergence thresholds were set to 1 × 10^−5^ eV for electronic self-consistency and 0.05 eV/Å for atomic forces. Effective U values of 4 eV for Fe and 5 eV for Cu were applied to account for the strong on-site Coulomb interactions of localized 3d electrons [19], [20]. Van der Waals interactions were considered using the DFT-D3 method with Grimme’s correction, and dipole corrections were applied to eliminate artificial surface effects and improve the accuracy of work function estimations.

## Result and discussion

### Characterizations of catalysts

**Fig. 1:**
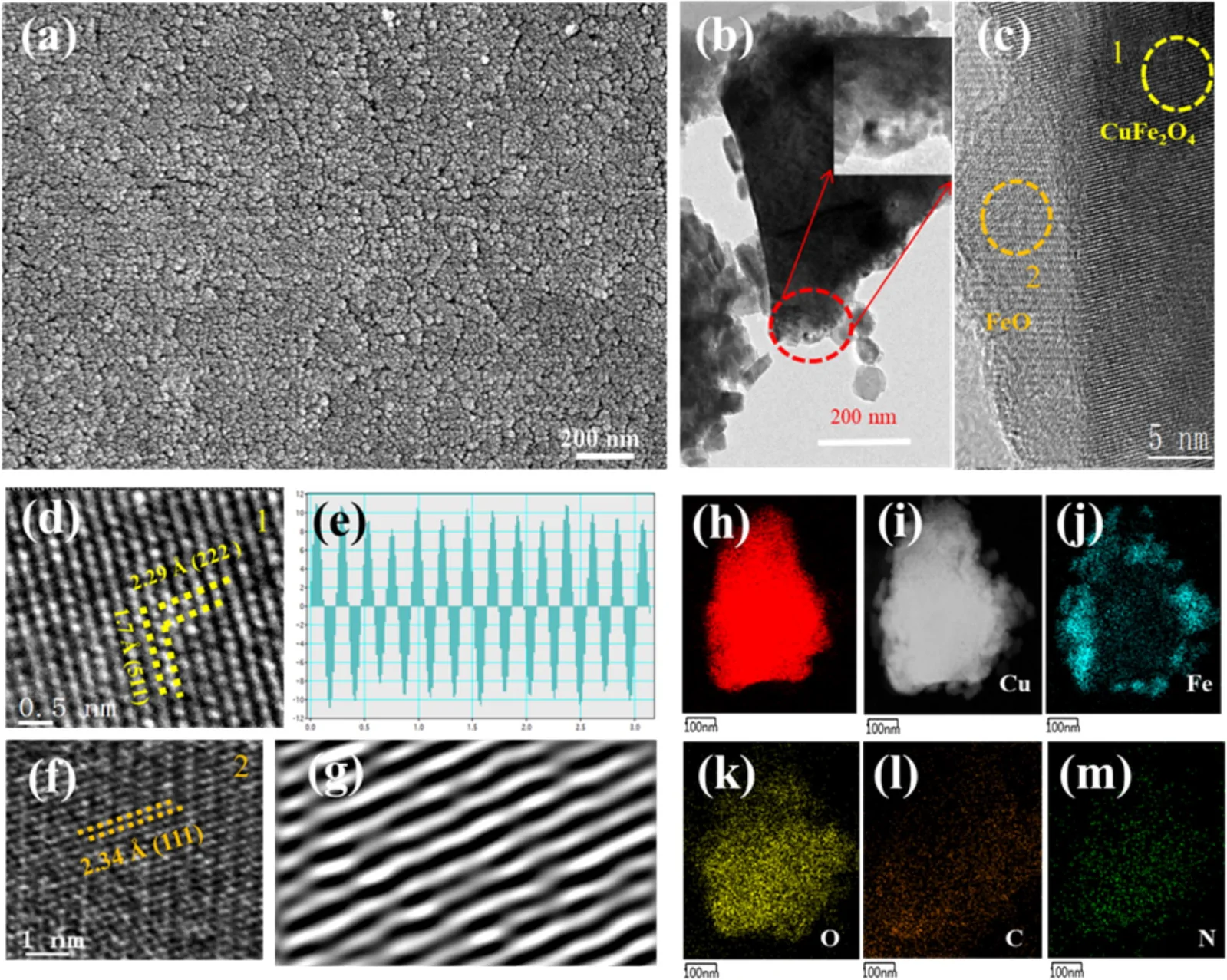
SEM and TEM characterization of the CFO/S-10 composite: (a) SEM image; (b) TEM image; (c) HRTEM image showing CuFe_2_O_4_ and FeO phases; (d, f) High-resolution lattice fringe images of CuFe_2_O_4_ (area 1) and FeO (area 2); (e) FFT pattern of lattice fringes; (g) Magnified view of lattice structure; (h) STEM-HAADF image; (i–m) Elemental mapping images of Cu, Fe, O, C, and N.

SEM, TEM, and HRTEM analyses were conducted to investigate the morphology and microstructure of the CFO/S-10 composite (Fig. 1). As shown in Fig. 1a and b, numerous nanostructured crystallites are uniformly anchored on the *S. palustre*-derived biochar matrix, forming a hierarchical architecture. These tightly coupled interfacial contacts are favorable for facilitating charge migration. The distinct contrast observed among nanoparticles suggests the coexistence of multiple crystalline phases and the formation of well-integrated heterojunctions. This configuration enhances the accessible surface area, provides abundant active sites for photocatalysis, and promotes multiple electron transport pathways within the composite [21].

The HRTEM image (Fig. 1c) reveals well-defined lattice fringes. In the magnified region (area 1), interplanar spacings of 2.29 Å and 1.70 Å are observed, corresponding to the (2 2 2) and (5 1 1) planes of CuFe_2_O_4_, respectively (Fig. 1d) [22]. The lattice fringes in area 2 are consistent with those of FeO (Fig. 1f) [23], confirming the coexistence of CuFe_2_O_4_ and FeO crystalline phases and the formation of a well-defined nanoscale heterojunction. The associated Fourier transform (FFT) patterns (Fig. 1e and g) further corroborate the crystalline features. EDS elemental mapping (Fig. 1h–m) indicates homogeneous dispersion of Cu and relatively denser Fe regions, further supporting the multiphase distribution. The presence of such intimately contacted interfaces promotes the generation of internal electric fields and asymmetric electron polarization, thereby enhancing charge separation and migration, ultimately improving photocatalytic efficiency [24].

**Fig. 2:**
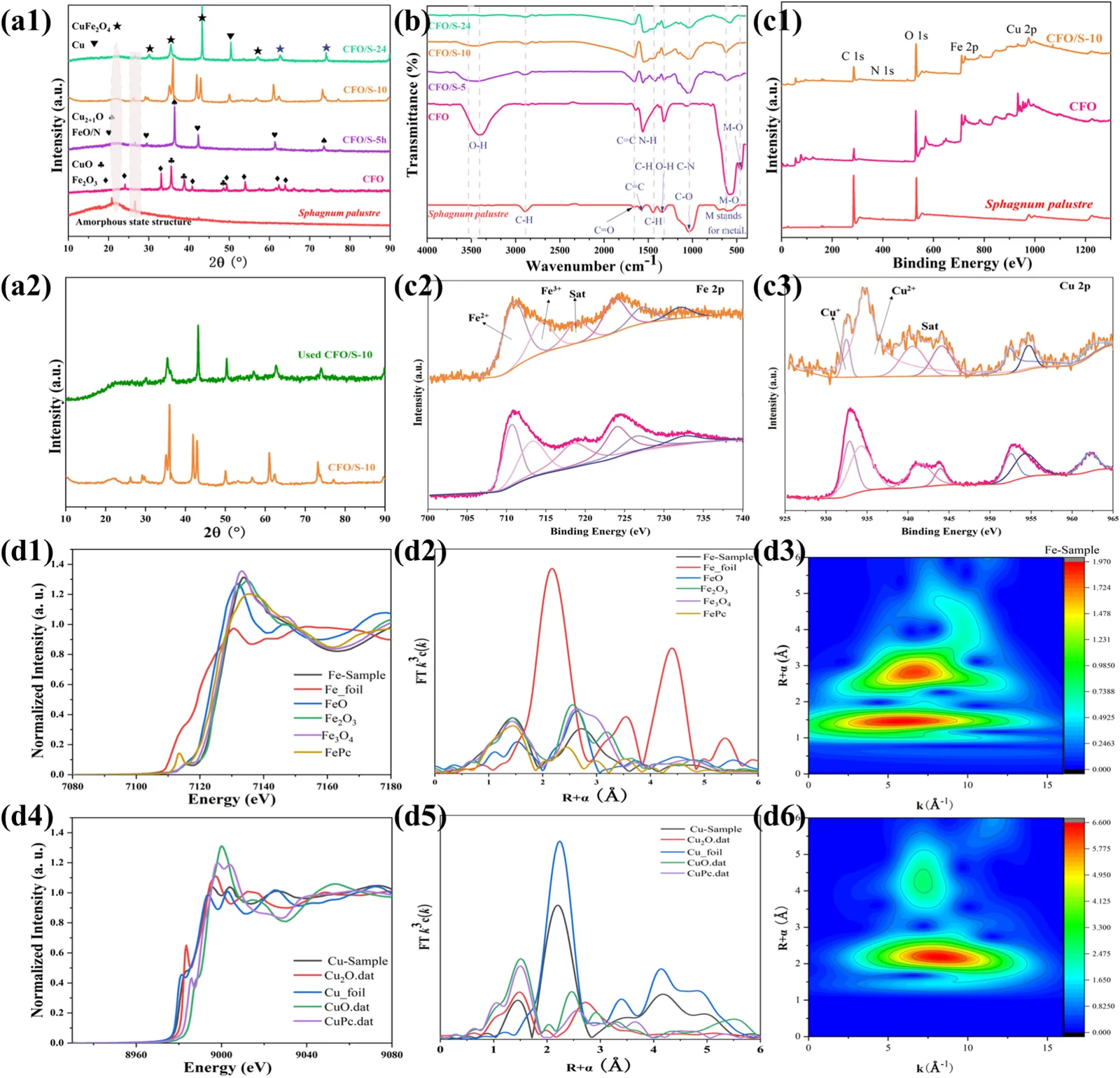
Structural and compositional characterization of the synthesized materials: (a1) XRD patterns of *S. palustre*, CFO, CFO/S-5, CFO/S-10, and CFO/S-24; (a2) XRD patterns of CFO/S-10 before and after the photocatalytic reaction; (b) FTIR spectra of *S. palustre*, CFO, CFO/S-5, CFO/S-10, and CFO/S-24; (c1) Full-range XPS spectra of *S. palustre*, CFO, and CFO/S-10; (c2, c3) High-resolution XPS spectra of Fe 2p and Cu 2p for CFO and CFO/S-10; (d1–d6). XANES and EXAFS analysis of Fe and Cu species in the CFO/S-10 composite: (d1, d4) Normalized XANES spectra of Fe and Cu with reference samples; (d2, d5) Fourier-transformed EXAFS spectra of Fe and Cu; (d3, d6) WT-EXAFS contour plots of Fe and Cu.

The XRD patterns of CFO and CFO/S samples are presented in Fig. 2a1. CFO/S-10 exhibits diffraction peaks characteristic of spinel CuFe_2_O_4_ and associated Fe and Cu oxides. Peak broadening and slight intensity reduction are attributed to the interaction between metal oxides and the amorphous carbon matrix. These results suggest that the abundant functional groups in *S. palustre* effectively coordinate with metal ions during hydrothermal synthesis, influencing crystal nucleation and the evolution of metal valence states through hydroxyl deprotonation and surface bonding interactions [25].

Fig. 2a2 shows the XRD patterns of CFO/S-10 sample before and after the photocatalytic reaction. After the reaction, the main peaks corresponding to CuFe_2_O_4_ remain clearly visible, indicating that the crystalline structure was preserved. However, the broad amorphous peaks originally attributed to the carbon matrix significantly decrease, likely due to structural rearrangement or coverage by adsorbed organic intermediates. Additionally, a slight increase in the intensity of the metallic Cu peak is observed after the reaction, possibly resulting from partial reduction or surface migration of Cu species under light irradiation. No new peaks or phase transitions were detected, confirming that the catalyst maintained excellent structural stability after the reaction.

The FTIR spectra (Fig. 2b) show that *S. palustre* contains abundant hydroxyl groups, hydrocarbon chains, and other oxygen-containing functional groups that serve as complexation sites for metal precursors [26], [27].

After formation of the composite, characteristic metal-oxygen stretching vibrations appear at approximately 670.7 cm^−1^, confirming the presence of Cu–O and Fe–O bonds. The peaks located at 2893.5 cm^−1^, 1447.6 cm^−1^, and 1345.1 cm^−1^ correspond to C–H stretching, C=C, and C–H bending vibrations, suggesting the retention of π-conjugated structures that can mediate electron transfer [28]. These functional groups may also enhance interfacial charge transport through improved carrier–carbon coupling.

To further probe the oxidation states and coordination environments [29], [30], XPS and XAS analyses were conducted (Fig. 2c, d, S1, and Table 1, Table 2). The O 1s spectrum (Fig. S1b) indicates that oxygen in CFO/S-10 is primarily in the form of metal–oxygen bonds, while the carbon matrix contributes C–O and C=O components (Fig. S1a) [31], [32].

The Fe 2p spectrum (Fig. 2c2) reveals the coexistence of Fe^2+^ and Fe^3+^, implying that the carbon matrix stabilizes low-valence Fe species [33]. The Cu 2p spectrum (Fig. 2c3) shows a mixture of Cu⁰, Cu^+^, and Cu^2+^ species, with a higher proportion of reduced Cu species in CFO/S-10 compared to CFO, indicating enhanced surface redox activity [34]. The shifts in binding energies further suggest strengthened metal–support interactions.

XANES analysis (Fig. 2d1 and d4) confirms that both Cu and Fe exist in mixed-valence states, with positions between the reference spectra of Cu_2_O/Cu and Fe_3_O_4_/Fe_2_O_3_. EXAFS spectra (Fig. 2d2 and d5) reveal coordination shells corresponding to Cu–O, Cu–Cu, Fe–O, and Fe–Fe bonds, suggesting that the local structure is well-preserved without forming large CuO aggregates [35], [36]. The wavelet transform results (Fig. 2d3 and d6) offer spatial–frequency resolution and validate Cu–O and Fe–O coordination environments. Quantitative fitting (Table 1, Table 2) indicates that Cu exhibits an average coordination number of 4.56 with a bond length of 1.85 Å, suggesting the presence of unsaturated sites that are favorable for catalytic reactions.

Collectively, SEM/TEM, FTIR, XRD, XPS, and XAS results demonstrate that CFO/S-10 features a multiphase heterojunction structure, with uniformly dispersed metal species, abundant surface functionalities, and mixed-valence Cu and Fe centers. This synergistic structure promotes the formation of internal electric fields and facilitates efficient charge transport, thereby enhancing electron–hole separation and the generation of reactive species, ultimately contributing to the material's superior photocatalytic degradation performance.

### Photoelectric properties and TCH removal mechanism

#### Photophysical properties and charge separation behavior

**Fig. 3:**
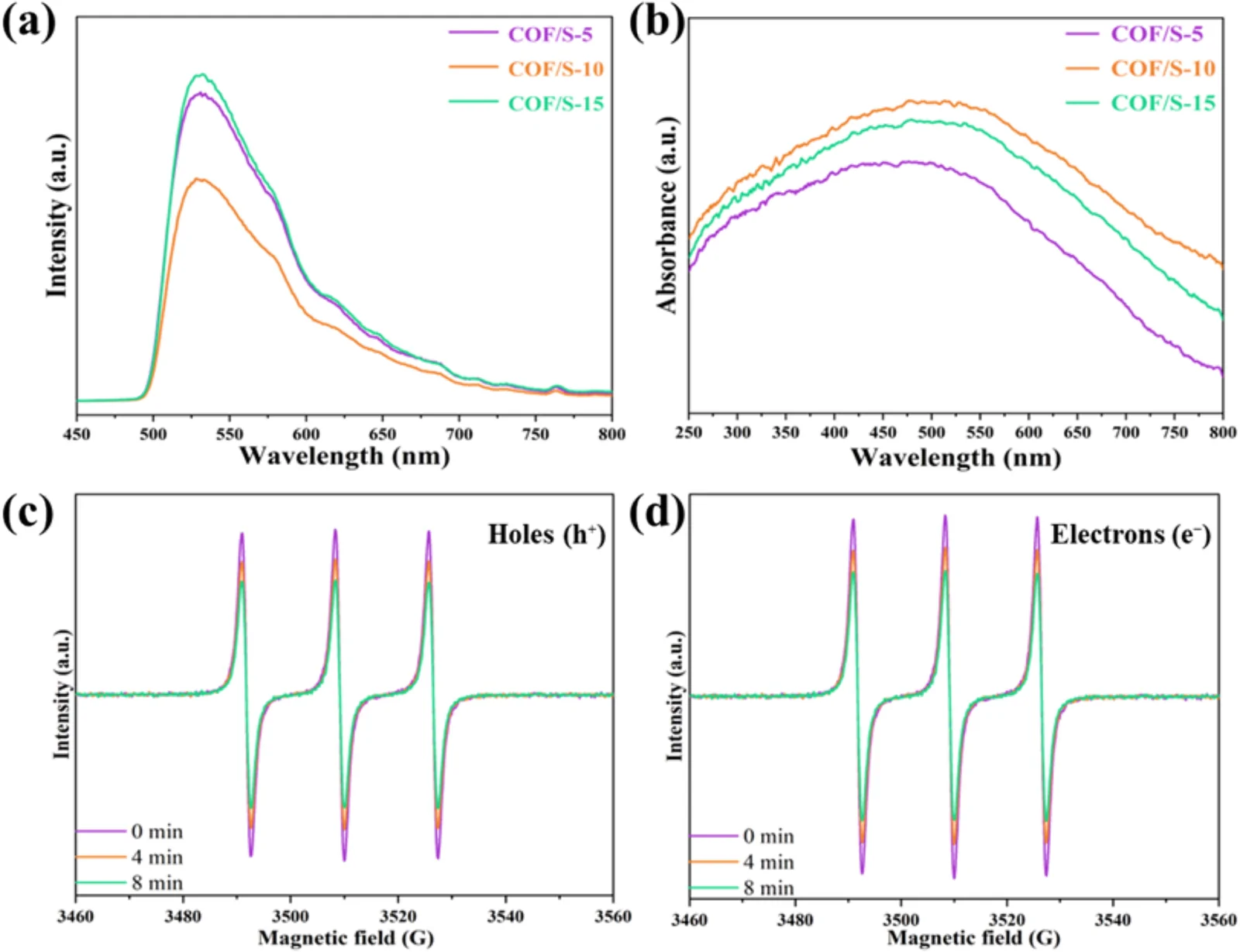
(a) and (b) PL and UV–Vis absorption spectra of CFO/S-5, CFO/S-10, and CFO/S-15 samples, respectively. (c-d) EPR spectra of •OH and •O_2_^−^ radicals for CFO/S-10 samples under visible light irradiation at 0, 4, and 8 min, respectively.

The photoelectric properties and charge carrier dynamics of the CFO/S composites were systematically investigated via PL, DRS, and EPR analyses. As shown in Fig. 3a, all samples exhibit a pronounced PL emission peak centered at approximately 530 nm. The PL intensity gradually decreases with increasing hydrothermal treatment time, indicating improved separation of photogenerated charge carriers [37]. This quenching behavior is primarily attributed to the formation of non-radiative recombination centers and the introduction of structural defects within the heterostructure. Specifically, the hydrothermal carbonization of *S. palustre* introduces abundant oxygen-containing functional groups and oxygen vacancies, while also facilitating the partial reduction of Cu^2+^ to Cu⁰/Cu^+^ and Fe^3+^ to Fe^2+^. These redox-active species and surface/bulk defects can introduce shallow mid-gap states that act as electron traps, effectively inhibiting radiative recombination and enhancing charge separation.

Furthermore, the built-in interfacial electric field formed between Cu/Fe oxides and the *S. palustre*-derived carbon matrix is expected to induce band bending, promoting interfacial charge separation and directional carrier transfer, possibly induced by interfacial electric fields. These synergistic effects of defect-induced charge trapping and interfacial electronic modulation are considered critical factors contributing to the enhanced photocatalytic performance.

To assess the optical harvesting capability, DRS analysis was performed over the 300–800 nm wavelength range. As shown in Fig. 3b, all samples show strong absorption in the 300–500 nm region, typical of CuFe_2_O_4_-based materials. Notably, the CFO/S-10 sample displays significantly stronger visible-light absorption than CFO/S-5, suggesting that prolonged hydrothermal treatment increases the density of optically active sites and improves light-harvesting efficiency. While the CFO/S-15 sample exhibits reduced absorption compared with CFO/S-10, this decline suggests that excessive hydrothermal treatment may lead to over-carbonization or structural densification, thereby weakening the light-harvesting capability.

EPR spectroscopy was employed to investigate the photogenerated charge carrier dynamics in CFO/S-10 under visible-light irradiation [38]. As shown in Fig. 3c and Fig. 3d, TEMPO (a stable nitroxyl radical) was used as a spin probe, and its characteristic EPR signals were monitored at 0, 4, and 8 min. A gradual decrease in TEMPO signal intensity was observed with increasing irradiation time, indicating its consumption through redox reactions with photogenerated electrons and/or holes.

The more pronounced quenching observed in the spectrum corresponding to the electron-trapping condition (Fig. 3d) suggests that electrons play a dominant role in the photocatalytic degradation, whereas holes contribute to a lesser extent (Fig. 3c). These results provide indirect evidence of efficient charge generation and separation within CFO/S-10.

Combined with PL quenching and enhanced DRS response, the EPR results suggest the presence of an efficient interfacial charge transfer mechanism. An S-scheme heterojunction is proposed, in which high-energy electrons from the CB of Fe/Cu oxides and holes from the VB of the carbonaceous phase are preserved for redox reactions, while low-energy carriers recombine at the interface, leading to enhanced photocatalytic activity.

#### Photocatalytic performance under different reaction conditions

The photocatalytic performance of the CFO/S composites was systematically evaluated using TCH as a model pollutant. Parameters such as the amount of *S. palustre*, hydrothermal treatment duration, solution pH, temperature, and light intensity were optimized to assess their impact on pollutant removal efficiency.

**Fig. 4:**
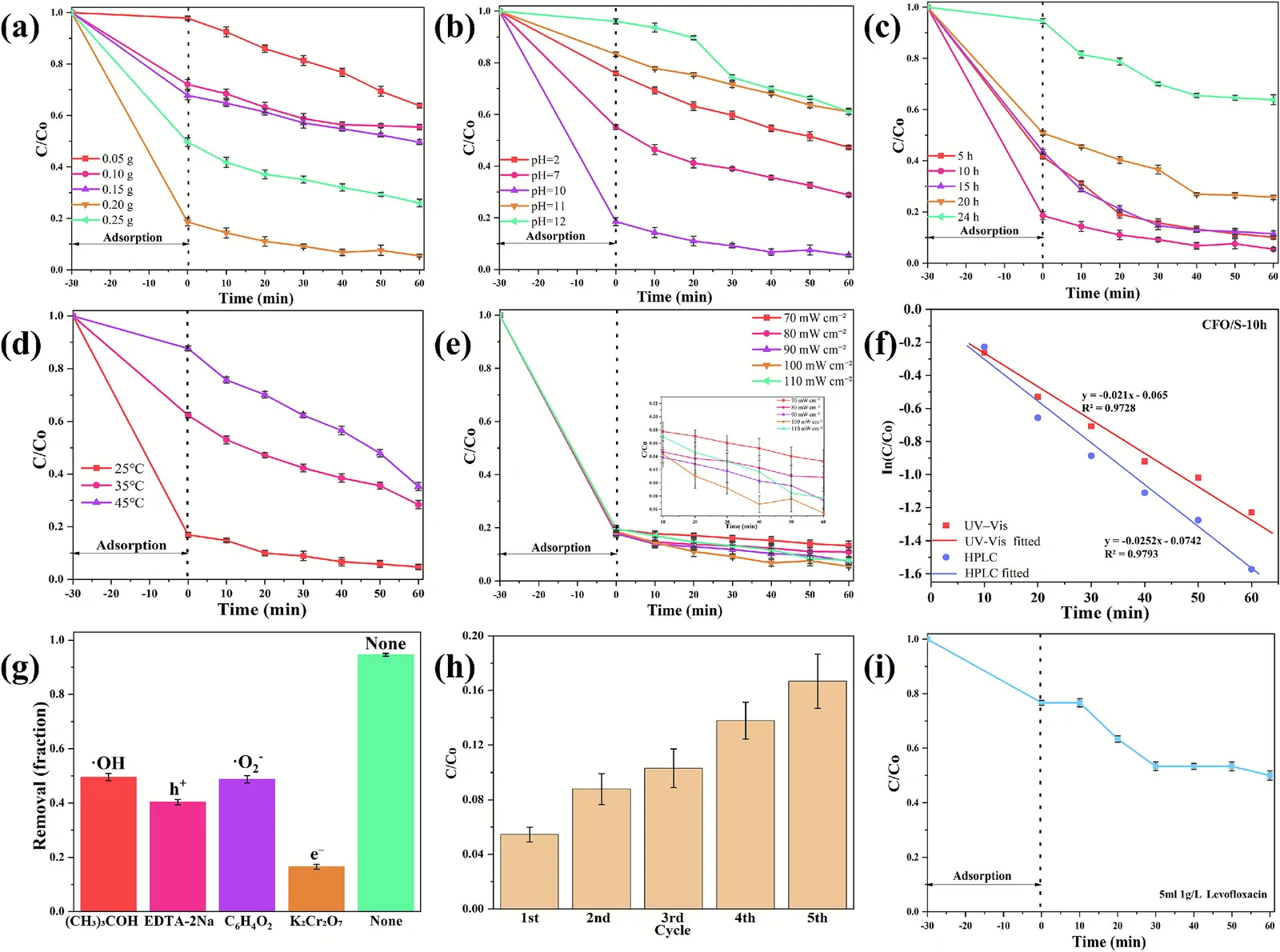
Photocatalytic degradation performance and stability of CFO/S samples under various conditions: (a) Effect of varying amounts of S. palustre (0.05–0.25 g); (b) Effect of initial pH (pH = 8–12); (c) Effect of hydrothermal reaction time (5–24 h); (d) Effect of reaction temperature (25–45 °C); (e) Effect of light intensity (70–110 mW cm^−2^); inset: enlarged view of the low-concentration region; (f) Pseudo-first-order kinetic plots of TCH degradation over CFO/S-10 h under visible light, evaluated by UV–Vis and HPLC analyses；(g) Effect of different radical scavengers on degradation efficiency; (h) Reusability of the CFO/S-10 sample over five consecutive photocatalytic cycles; (i) Photocatalytic degradation of LVX by CFO/S-10 under visible-light irradiation.

As illustrated in Fig. 4a, the degradation efficiency initially increased and then decreased with the gradual increase in *S. palustre* content from 0.05 to 0.25 g, reaching a maximum at 0.20 g. This optimum loading is mainly attributed to the synergistic effects between CuFe_2_O_4_ and the carbonaceous matrix, which enhance light absorption, charge carrier generation, and interfacial separation. However, excessive biochar content induces light scattering and surface shielding effects, thereby reducing photon penetration and limiting access to active catalytic sites. These results underscore the importance of balancing carbon content to ensure efficient interfacial interactions within the composite structure [39].

As shown in Fig. 4b, photocatalytic performance was also strongly dependent on hydrothermal treatment duration. The CFO/S-10 sample exhibited the highest degradation efficiency, owing to its improved crystallinity and well-developed internal electric field, which facilitate directional charge transport and suppress recombination. In contrast, insufficient hydrothermal treatment (e.g., 5 h) led to poor structural development, while excessive treatment (e.g., 15 h) caused grain coarsening and partial encapsulation of active sites, thus reducing catalytic activity.

The influence of initial solution pH on the degradation of TCH is depicted in Fig. 4c. Optimal performance was achieved under weakly alkaline conditions (pH = 10), where the catalyst surface potential approximates its point of zero charge, thereby favoring the adsorption of TCH molecules and the generation of ROS (•OH and •O_2_^−^). Under acidic conditions (pH = 2), surface protonation suppresses both adsorption and ROS generation, leading to diminished degradation efficiency. At neutral pH, intermediate activity was observed, whereas at strongly alkaline pH, side reactions and reduced ROS stability slightly impaired performance.

As illustrated in Fig. 4d, the influence of temperature on TCH degradation further confirms that the removal process is predominantly governed by adsorption rather than photocatalysis. A gradual decrease in TCH removal efficiency was observed with increasing temperature, consistent with the exothermic nature of adsorption. Elevated temperatures reduce adsorption capacity, thereby limiting subsequent photochemical degradation.

As shown in Fig. 4e, increasing the light intensity enhances the degradation efficiency, reaching a maximum at 100 mW cm^−2^. Beyond this threshold, a slight decline in performance was observed, likely due to the saturation of active sites, thermal accumulation effects, and increased electron–hole recombination. These trends support an adsorption-modulated photocatalytic mechanism, in which the pre-concentration of pollutants on the catalyst surface is critical for subsequent light-driven degradation.

As shown in Fig. 4f, the photocatalytic degradation of TCH over CFO/S-10 follows pseudo-first-order kinetics. Based on UV–Vis absorption data, the apparent rate constant (k) was calculated to be 0.021 min^−1^ with a correlation coefficient (R^2^) of 0.9728. To validate the reliability of this result, HPLC analysis was conducted for the same reaction system. The HPLC-derived kinetic fitting yielded a slightly higher rate constant of 0.0252 min^−1^ with R^2^ = 0.9793, further confirming the consistent degradation behavior. This deviation is attributed to the fact that single-wavelength UV–Vis spectroscopy may capture residual absorbance from degradation intermediates or light scattering by photocatalyst particles [40], whereas HPLC specifically quantifies the parent TCH molecule with higher accuracy.

Although the rate constant is slightly lower than those reported for other biochar-based photocatalysts, such as MOF-derived biochar composites (0.03478 min^−1^) [41], hollow biochar sphere-supported TiO_2_ (0.0301 min^−1^) [42], and CaIn_2_S_4_-ZnO/pine cone-derived biochar (0.094 min^−1^) [43], it remains comparable to Ag-doped biochar/g-C_3_N_4_/TiO_2_ (0.0207 min^−1^) [44], as summarized in Table 1. Notably, the removal of TCH by CFO/S-10 is predominantly governed by adsorption, while photocatalysis plays a secondary, synergistic role. Thus, the rate constant reflects the combined contribution of both processes. Despite this, CFO/S-10 exhibits excellent overall removal efficiency and demonstrates strong potential for practical wastewater treatment applications.

To further elucidate the degradation mechanism, the roles of reactive species were investigated through radical quenching experiments, as shown in Fig. 4g. The addition of K_2_Cr_2_O_7_, a selective electron scavenger, resulted in the most pronounced suppression of activity, indicating the crucial role of photogenerated electrons. Substantial declines in activity were also observed with (CH_3_)_3_COH (•OH scavenger) and C_6_H_4_O_2_ (•O_2_^−^ scavenger), by 49.6% and 48.7%, respectively, suggesting that both hydroxyl and superoxide radicals significantly contribute to oxidation process. In contrast, moderate inhibition by EDTA-2Na (hole scavenger) implies a secondary role for photogenerated holes. These results are consistent with the EPR analysis and further validate the proposed degradation mechanism.

The reusability of the CFO/S-10 photocatalyst was assessed over five successive TCH degradation cycles, as shown in Fig. 4h. Although a gradual reduction in performance, the catalyst maintained approximately 83.32% of its initial efficiency after five cycles. This slight decline is attributed to catalyst loss during recovery, surface fouling by residual intermediates, and partial deactivation of active sites. Nonetheless, the catalyst demonstrated favorable structural stability and recyclability, underscoring its potential for long-term application in water purification.

Selectivity of the CFO/S-10 photocatalyst was further evaluated using levofloxacin (LVX) as a comparison pollutant, as shown in Fig. 4i. Under identical conditions, only about 50% of LVX was removed, much lower than that of TCH (94.56%), indicating structure-dependent selectivity. The higher removal of TCH can be attributed to its abundant polar and ionizable groups, which facilitate stronger interaction with the oxygen-rich biochar matrix and promote adsorption-driven photocatalytic degradation. In contrast, the more hydrophobic structure of LVX leads to weaker adsorption and reduced degradation efficiency.

To further assess the performance of the CFO/S-10 photocatalyst, a comparative analysis was conducted against representative biochar-based materials reported for the visible-light-driven degradation of TCH, as summarized in Table 3. Although the CFO/S-10 does not exhibit the highest degradation rate among reported systems, it achieves a competitive removal efficiency of 94.56% and maintains stable performance over repeated cycles, highlighting the robustness of the material design.

**Table 3:** Summary of representative biochar-based photocatalysts for visible-light degradation of TCH.

Photocatalyst (Biocchar Source)	Light	TCH concentration (mg⋅L^−1^)	Catalyst dosage (mg or mg·L^−1^)	Time (min)	Efficiency	Rate constant (min^−1^)	Reusability	Reference
NSCM-20 biochar (Cow dung)	300 W xenon lamp	40	200 mg·L^−1^	120	94.62%,	0.03478	5 cycles, >80%	[41]
TiO_2_/Fe-Cu-HBS composite biochar (Starch)	500 W xenon lamp 100 mW·cm^−2^	30	500 mg·L^−1^	120	95.4%	0.0301	5 cycles, 91.5%	[42]
CaIn_2_S_4_–ZnO/biochar composite (Pine cone waste)	300 W Xenon lamp	50	1000 mg·L^−1^	80	>95%	0.094	6 cycles, >86%	[43]
Ag-doped biochar/g-C_3_N_4_/TiO_2_ composite (Cotton fiber waste)	300 W xenon lamp, λ ≥ 420 nm	20	250 mg·L^−1^	80	90.4%	0.0207	——	[44]
La_2_S_3_/S,N co-doped biochar (Sugarcane bagasse)	Xe lamp, 400 mW·cm^−2^	200	400 mg·L^−1^	120	98.45%	——	5 cycles, 93.26%	[45]
Biochar-supported m-BiVO_4_ (Yeast powder template)	500 W xenon lamp, λ ≥ 420 nm	10	30 mg	180	89.9%	——	5 cycles, >70%	[46]
Er^3+^-doped BiOCl-loaded biochar (Bamboo leaves)	300 Wxenon lamp, λ ≥ 420 nm,600 nm	20	500 mg·L^−1^	90	89.2%	——	5 cycles, nearly unchanged	[47]
3D biochar/g–C_3_N_4_ composite (Wood flour)	250 W Xenon lamp, λ = 420 nm	10	20 mg	60	98.04%	——	4 cycles, nearly unchanged	[48]
CFB − BWO 2D/2D biochar (Cotton fibers)	500Wxenon lamp with a 420 nm cut-off filter	20	250 mg·L^−1^	360	96.8%	0.2681	5 cycles, nearly unchanged	[49]
Red mud-based biochar (rice husk)	300 W	20	80 mg·L^−1^	60	99.5%	——	5 cycles, >95%	[50]
BiFeO_3_/ biochar (Kenaf fiber)	300 W xenon source, λ > 400 nm	50	200 mg·L^−1^	200	>95%	0.02761	——	[51]
CFO/S-10 (*Sphagnum palustre*)	300 W xenon lamp	50	200 mg·L^−1^	60	94.56%	0.021	5 cycles, 83.32%	This work

In addition, the photocatalytic performance of CFO/S-10 was compared with various Cu–Fe oxide-based catalysts reported for the degradation of antibiotic degradation (see Table S1). While CFO/S-10 may not surpass all conventional Cu–Fe photocatalysts in degradation efficiency, its biochar-assisted architecture imparts several distinct advantages, including enhanced adsorption affinity, improved material sustainability, and facile recovery. These features make CFO/S-10 a competitive and environmentally viable candidate for practical wastewater treatment applications, particularly in systems requiring both adsorption and photocatalysis.

#### Identification of degradation intermediates and reaction pathways

**Fig. 5:**
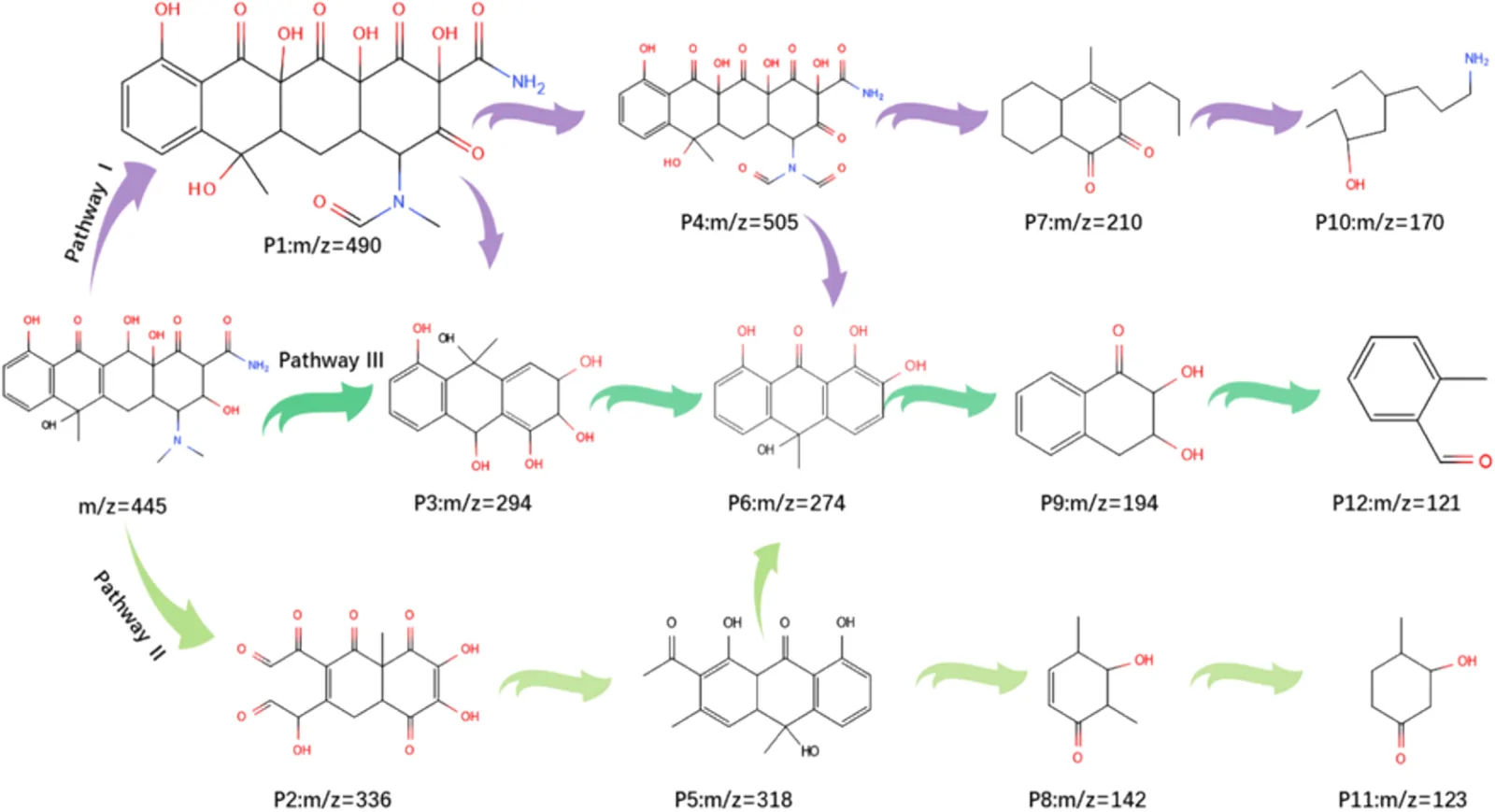
Schematic diagram of the degradation pathways for the products of the CFO/S-10 sample after 60 min of photocatalytic reaction, along with the structures and molecular weights of the main intermediate products.

To elucidate the photocatalytic degradation mechanism of TCH, the intermediates products formed after 60 min of visible-light irradiation were analyzed using HPLC–MS. A total of twelve degradation products were identified. Based on their molecular ion peaks, fragmentation patterns, and comparisons with previously reported data [52], [53], three main degradation pathways (designated as I, II, and III) were proposed, as illustrated in Fig. 5.

Pathway I involves an initial electrophilic attack by ROS, primarily •OH and h^+^, on the parent TCH molecule (P1, *m*/*z* = 490). This leads to hydroxylation and oxidation of the aromatic rings, forming hydroxylated intermediates such as P4 (*m*/*z* = 505), followed by demethylation and progressive ring cleavage. These steps generate smaller products including P7 (*m*/*z* = 210) and P10 (*m*/*z* = 170), which indicate significant fragmentation of the TCH backbone.

Pathway II is initiated by hydroxylation of the aromatic side chain of TCH, resulting in the formation of P2 (*m*/*z* = 336). This is followed by successive oxidation and structural transformation, producing intermediates such as P5 (*m*/*z* = 318) and P6 (*m*/*z* = 274), both exhibiting partial ring-opening features. Further oxidative cleavage of P6 leads to low-molecular-weight products, including P9 (*m*/*z* = 194) and P12 (*m*/*z* = 121), suggesting progressive mineralization. Additionally, the appearance of P8 (*m*/*z* = 142) and P11 (*m*/*z* = 123) suggests parallel fragmentation routes originating from P5. These small-ring structures represent late-stage degradation intermediates, which are readily mineralized into CO_2_ and H_2_O.

Pathway III involves demethylation and rearrangement of methyl-substituted intermediates. Specifically, P3 (*m*/*z* = 294) is generated through demethylation and ring rearrangement, followed by oxidative cleavage to form P8 (*m*/*z* = 142) and P11 (*m*/*z* = 123), which are further mineralized into CO_2_ and H_2_O [54], [55].

Collectively, the photocatalytic degradation of TCH proceeds via multiple transformation reactions, including hydroxylation, dechlorination, demethylation, and ring-opening, ultimately converting the parent compound into less toxic intermediates. Under prolonged visible-light irradiation, these intermediates are expected to undergo complete mineralization to CO_2_ and H_2_O. The structures and corresponding *m*/*z* values of all identified intermediates are summarized in Table S2.

The detection of various oxidative intermediates and their sequential transformation pathways confirms the high redox capability of the CFO/S-10 composite during the photocatalytic degradation process. These results further substantiate the proposed charge-transfer mechanism and highlight the system’s capability to achieve effective mineralization of recalcitrant antibiotic pollutants under visible-light irradiation.

### First-principles calculations

**Fig. 6:**
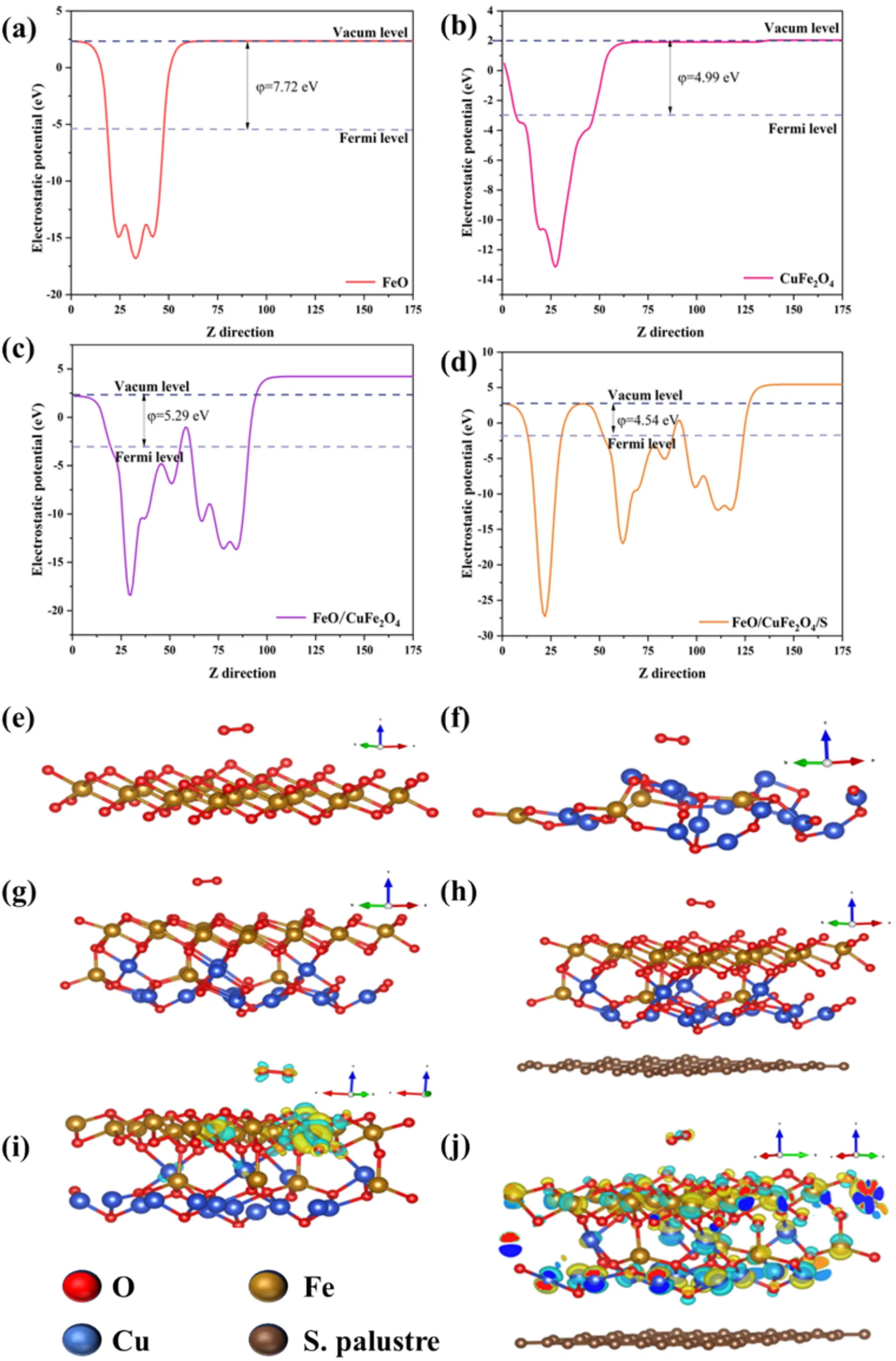
(a–d) Work functions of FeO, CuFe_2_O_4_, FeO/CuFe_2_O_4_, and FeO/CuFe_2_O_4_/S; (e–h) Adsorption configurations of O_2_ molecules on the surfaces of the corresponding materials; (i, j) Electron density distributions of FeO/CuFe_2_O_4_ and FeO/CuFe_2_O_4_/S.

To gain atomic-scale insight into the interfacial charge transfer behavior and validate the proposed S-scheme mechanism, first-principles calculations based on DFT were conducted. The work functions, oxygen adsorption configurations, and differential charge density distributions are summarized in Fig. 6 and Table 4.

**Table 4:** Theoretical comparison of work function and O_2_ adsorption energy for different structures.

Sample	Vacuum potential (eV)	Fermi level (eV)	Work function (eV)	Adsorption energy (eV)
FeO	2.34	−5.38	7.72	−2.27
CuFe_2_O_4_	2.04	−2.96	4.99	−1.21
FeO/CuFe_2_O_4_	2.21	−3.08	5.29	−4.01
FeO/CuFe_2_O_4_/S	2.82	−1.72	4.54	−1.12

As shown in Fig. 6a–d and Table 4, FeO exhibits a relatively high work function of 7.72 eV, indicating strong electron confinement and inherently low electron transfer capability. In comparison, CuFe_2_O_4_ displays a lower work function of 4.99 eV, implying more accessible surface electrons. After constructing the FeO/CuFe_2_O_4_ heterojunction, the work function decreases to 5.29 eV, suggesting enhanced interfacial electronic coupling and favorable charge redistribution. Further integration with *S. palustre*-derived biochar reduces the work function to 4.54 eV, demonstrating that the carbonaceous matrix facilitates electron delocalization and decreases the potential barrier for carrier transport. This stepwise decline in work function reflects interfacial charge redistribution and band bending, consistent with an S-scheme mechanism where low-energy carriers recombine across the interface, while high-energy electrons in FeO are preserved for reduction reactions.

The optimized O_2_ adsorption configurations on different surfaces are displayed in Fig. 6e–h. On FeO, O_2_ molecules retain a nearly linear geometry, indicating weak physisorption. In contrast, adsorption on CuFe_2_O_4_ results in a bent O_2_ configuration, reflecting enhanced activation due to the cooperative catalytic roles of Cu and Fe. The FeO/CuFe_2_O_4_ heterojunction further amplifies this effect, as evidenced by pronounced O–O bond deformation. Although the FeO/CuFe_2_O_4_/S composite exhibits a lower oxygen adsorption energy (−1.12 eV) compared with FeO/CuFe_2_O_4_ (−4.01 eV), the adsorbed O_2_ molecule still shows a pronounced deviation from linear geometry (Fig. 6h), indicating the retention of catalytic activity and effective O_2_ activation.

The spatial charge redistribution is depicted in Fig. 6i and j. In the FeO/CuFe_2_O_4_ system, electron accumulation is observed around the adsorbed O_2_, while electron depletion is localized near the Fe and Cu centers. This distribution indicates effective charge polarization between the active metal sites and the adsorbates, facilitating catalytic activation. In the FeO/CuFe_2_O_4_/S composite, a more delocalized charge accumulation region is observed, indicating enhanced interfacial polarization and accelerated charge migration. The biochar matrix contributes in two key ways: it strengthens the built-in electric field and improves the transport efficiency of photogenerated carriers to catalytic sites.

Taken together, the progressive decrease in work function, enhanced O_2_ activation behavior, and strengthened interfacial charge redistribution collectively confirm the formation of a S-scheme heterojunction within the FeO/CuFe_2_O_4_/S system. In this architecture, high-energy electrons in FeO and high-energy holes in CuFe_2_O_4_ are spatially preserved to participate in redox reactions, while low-energy carriers recombine across the interface. This selective charge migration pathway maintains strong redox potential while effectively suppressing carrier recombination, thereby underpinning the superior photocatalytic performance observed for CFO/S.

### Mechanism analysis

**Fig. 7:**
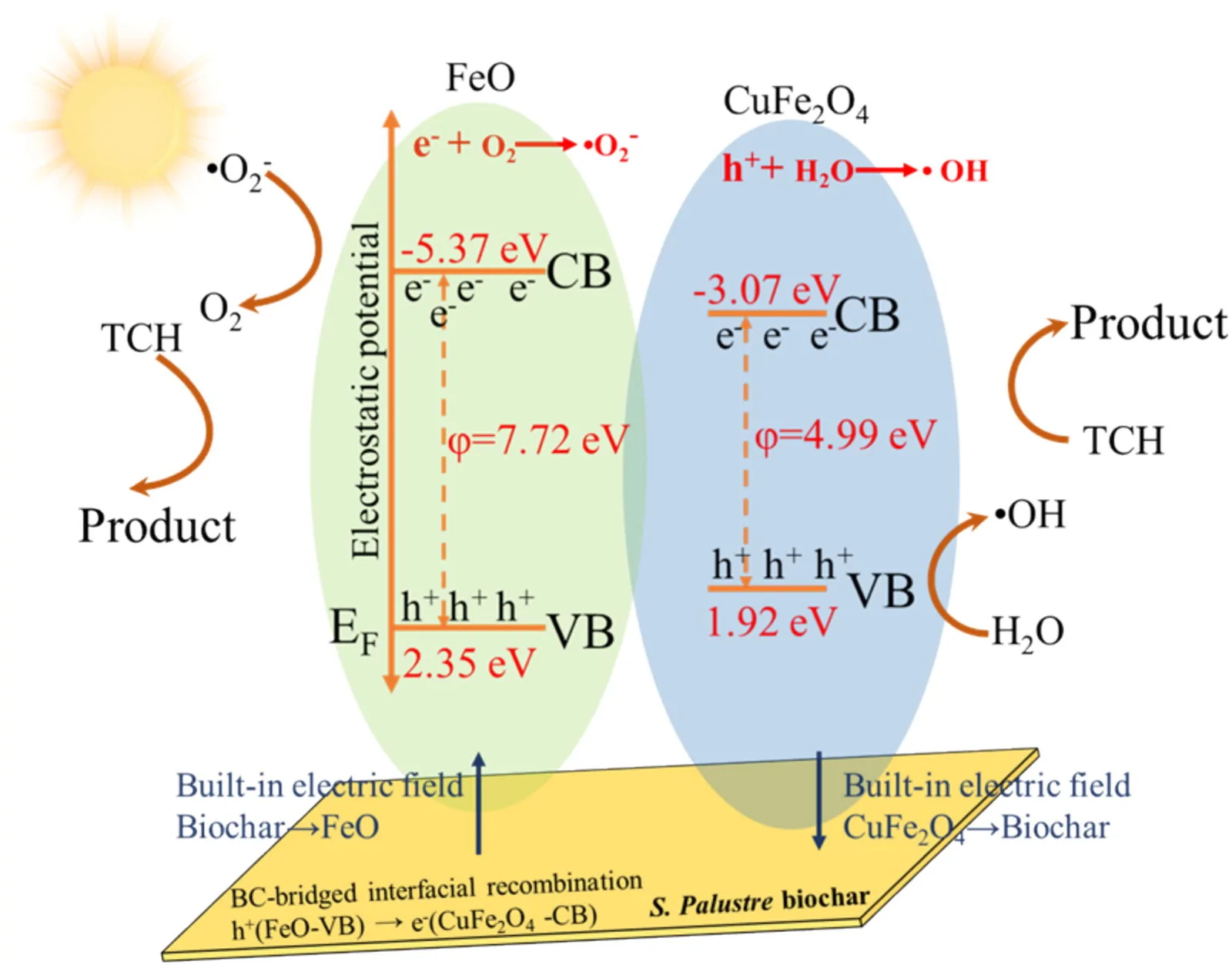
Photocatalytic mechanism of TCH degradation.

The CFO/S composite exhibits significantly enhanced removal efficiency toward TCH, which is predominantly governed by adsorption, with additional contributions from visible-light-driven photocatalysis. Based on experimental results, degradation intermediates, first-principles calculations, and spectroscopic analyses, a photocatalytic reaction mechanism is proposed and illustrated in Fig. 7, focusing specifically on the light-induced degradation pathway.

As the dominant process, the porous biochar matrix derived from *S. palustre* provides abundant oxygen-containing functional groups and π–π interaction sites, enabling rapid adsorption and local enrichment of TCH molecules at the catalytic interface. This adsorption-driven concentration effect significantly enhances the photocatalytic degradation efficiency by increasing the availability of reactants near active redox sites.

Upon visible-light excitation, an S-scheme heterojunction is formed between FeO and CuFe_2_O_4_, facilitating spatial separation and directional migration of photogenerated charge carriers. The recombination of low-energy electrons in the CB of CuFe_2_O_4_ with low-energy holes in the VB of FeO allows for the preservation of high-energy electrons in FeO and high-energy holes in CuFe_2_O_4_. Although the contribution of this pathway is secondary to that of adsorption, it plays a critical role in pollutant oxidation. The retained electrons reduce O_2_ to generate •O_2_^−^, while the holes oxidize water or hydroxyl species to yield •OH. These ROS collaboratively attack adsorbed TCH molecules via stepwise oxidative transformations, such as hydroxylation, demethylation, and ring opening, eventually achieving partial to deep mineralization into CO_2_ and H_2_O, as evidenced in Fig. 5.

Furthermore, the coexistence of Cu(I)/Cu(II) and Fe(II)/Fe(III) redox pairs enhances interfacial polarization and accelerates charge migration across the biochar-supported heterostructure. This is substantiated by DFT results, which reveal a reduced work function and improved O_2_ activation capability upon biochar incorporation (Table 3). While photocatalysis is not the dominant removal route, it significantly improves degradation kinetics and enhances the overall detoxification process.

In summary, the removal of TCH by the CFO/S composite proceeds via a hybrid adsorption–photocatalysis mechanism, where: (i) *S. palustre*-derived biochar governs adsorption, serving as the primary pathway for pollutant capture; (ii) The S-scheme photocatalytic process facilitates ROS-mediated oxidation of adsorbed species, contributing to detoxification and mineralization; (iii) The heterostructure’s interfacial coupling and redox-active centers promote selective carrier transfer and sustain strong redox potential.

This cooperative mechanism underscores the potential of CFO/S as a highly efficient, adsorption-dominated catalyst with photocatalytic enhancement, offering both rapid pollutant removal and green remediation under visible-light conditions.

## Conclusions

In this study, a visible-light-responsive heterojunction photocatalyst was successfully constructed by integrating *S. palustre*-derived biochar with Cu–Fe bimetallic oxides (CFO/S) via hydrothermal synthesis. The synergistic interaction between Cu and Fe oxides and the formation of a FeO/CuFe_2_O_4_/S heterostructure significantly enhanced the photogenerated charge carrier separation and facilitated the desorption of surface-bound intermediates. These improvements collectively contributed to the efficient removal of TCH, which was primarily governed by adsorption, with photocatalytic degradation serving as a complementary pathway under visible-light irradiation.

Among all tested samples, the CFO/S-10 composite exhibited the highest removal efficiency, achieving approximately 94.56%. This superior performance was attributed to its optimized crystallinity, enhanced internal electric field, and high interfacial adsorption sites. The abundant oxygen-containing functional groups in the *S. palustre*-derived carbon matrix significantly promoted TCH adsorption at the solid–liquid interface. They also played a crucial role in anchoring metal oxides and preserving the structural integrity of the composite during repeated photocatalytic cycles.

Mechanistic studies revealed that photogenerated e^−^ were the dominant reactive species responsible for oxidative degradation, while •OH, •O_2_^−^, and h^+^ also contributed to the degradation pathway. These species sequentially attacked TCH via hydroxylation, demethylation, and ring-opening reactions, ultimately leading to mineralization into CO_2_ and H_2_O. The proposed S-scheme charge transfer mechanism was corroborated by first-principles calculations and degradation product analysis.

Although all photocatalytic tests were conducted under controlled laboratory conditions, the findings highlight the promising potential of CFO/S materials for practical water treatment applications. Future work will focus on evaluating their performance in real wastewater systems containing diverse coexisting ions and organic pollutants.

In summary, the CFO/S-10 composite represents a sustainable, cost-effective, and efficient platform for visible-light-driven antibiotic removal, offering valuable insights into the rational design of biochar-based heterojunction materials for environmental remediation.

## Declaration of competing interest

The authors declare that they have no known competing financial interests or personal relationships that could have appeared to influence the work reported in this paper.
