# Supplementary material for: Cu/Fe co-doped Sphagnum palustre-derived biochar for the synergistic adsorption and photocatalytic removal of tetracycline hydrochloride
Source: J Adv Res. 2025 Dec 29;87:405–18. doi: 10.1016/j.jare.2025.12.051 (PMC13539320; doi:10.1016/j.jare.2025.12.051)
Supplement: Supplementary file 1 [file mmc1.docx]

**Supplementary Material**

**Cu/Fe co-doped *Sphagnum palustre*-derived biochar for the synergistic adsorption and photocatalytic removal of tetracycline hydrochloride**

**Figures and Tables**

**Fig. S1.** XPS spectra of (f) C 1s and (g) O 1s for *S. palustre*, CFO, and CFO/S-10 samples, respectively.

**Table S1.** Summary of representative photocatalysts for visible-light-driven degradation of antibiotics.

| Photocatalyst | Light | Antibiotic concentration  (mg·L⁻¹) | Catalyst dosage (mg or mg·L⁻¹) | Time (min) | Efficiency | Rate  constant  (min^- 1^) | Reusability | Reference |
| --- | --- | --- | --- | --- | --- | --- | --- | --- |
| PANI/CuFe_2_O_4_ composite | 500-watt halogen lamp | 50 (TCH) | 100 mg | 120 | 86% | 0.014 | 4 cycles, 81% | [1] |
| CuFe_2_O_4_/ In_2_S_3_ composite | 300 W Xenon lamp | 30 (TCH) | 50 mg | 150 | 95.2% | 0.0316 | —— | [2] |
| ZIF-8/g-C_3_N_4/_  CuFe_2_O_4_ composite | 50 W LED lamp,  λ> 420 nm | 15 (TC) | 750 mg·L⁻¹ | 65 | 99.15% | 0.0484 | 4 cycles, a small decrease | [3] |
| g-C_3_N_4_/TiO_2_/CuFe_2_O_4_ omposite | visible light using LED lamp | 20.01 (TC) | 13.99 mg | 40 | 98.47 % | 0.1013 | 5 cycles, no significant decrease | [4] |
| CuFe_2_O_4_/ABC biochar | Visible light | 30 (TC) | 200 mg·L⁻¹ | 150 | 94.89 % | 0.017 | 5 cycles, 88.02% | [5] |
| Cu_2_O@Fe_3_O_4_ composite | Visible light | 20 (TC) | 60 mg·L⁻¹ | 30 | 97.67% | —— | 5 cycles, 93.3% | [6] |
| CuFe_2_O_4_-SnS_2_ composite | 300 W halogen lamp, λ*>* 400 nm | 20 (TC) | 0.2 mg·L⁻¹ | 60 | 98% | 0.04283 | 6 cycles, a minor reduction | [7] |
| CuFe_2_O_4_/C/SiO_2_/PMS composite | 300 W Xe lamp | 20 (TC) | 10 mg | 15 | 98.41% | 0.076 | 4 cycles, 76.32% | [8] |
| CuO/Fe_3_O_4_/GO composite | —— | 30 TC | 200 mg·L⁻¹ | ～140 | 97.3% | 0.024 | 5 cycles, 91.3% | [9] |
| Cu_2_O/Fe_3_O_4_/MIL-101(Fe) composite | 500 W  Xe lamp | 20 (Cprofloxacin) | 500 mg·L⁻¹ | 105 | 99.2% | 0.04 | 5 cycles, 6% reduction | [10] |
| CFO/S-10 | 300 W xenon lamp | 50 (TCH) | 200 mg·L⁻¹ | 60 | 94.56% | 0.021 | 5 cycles, 83.32% | This work |

**Table S2.** Identified intermediates of TCH photocatalytic degradation by CFO/S-10 from HPLC–MS analysis and their proposed pathways.

| m/z | Intermediate ID | Proposed structural change / feature | Pathway |
| --- | --- | --- | --- |
| 490 | P1 | Parent TCH molecule | I |
| 505 | P4 | Hydroxylation and oxidation (–OH, C=O introduced) | I |
| 210 | P7 | Demethylation and progressive ring cleavage product | I |
| 170 | P10 | Extensive fragmentation of tetracycline backbone | I |
| 336 | P2 | Hydroxylation on aromatic side chain | II |
| 318 | P5 | Oxidation and partial ring-opening | II |
| 274 | P6 | Oxidative ring-opening intermediate | II |
| 194 | P9 | Ring-cleavage product (low-molecular-weight) | II |
| 121 | P12 | Final ring-opening product (deep oxidation) | II |
| 294 | P3 | Demethylation and rearrangement of methyl-substituted TCH | III |
| 142 | P8 | Fragment from P5 or P3 oxidation/ring-opening | II / III |
| 123 | P11 | Fragment from P5 or P3 oxidation/ring-opening | II / III |

**Reference**

[1] Gaffar, A. Kumar, J. Alam, U. Riaz, Efficient visible light–induced photocatalytic degradation of tetracycline hydrochloride using CuFe₂O₄ and PANI/CuFe₂O₄ nanohybrids, Environmental Science and Pollution Research 30 (2023) 108878–108888.

[2] Z. Chen, T. Su, X. Luo, Z. Qin, H. Ji, Preparation of CuFe₂O₄/In₂S₃ composite for photocatalytic degradation of tetracycline under visible light irradiation, Reaction Kinetics, Mechanisms and Catalysis 137 (2024) 587–606.

.[3] A. Kumar, A.M. Hussein, F.M.A. Altalbawy, M. Kaur, H. Kaur, S. Salah Jalal, S. Hassan Zain Al-Abdeen, K. Muzammil, M. Alhadrawi, Synthesis of g-C₃N₄ decorated with ZIF-8 and CuFe₂O₄ as a highly efficient and magnetically separable photocatalyst for degradation of tetracycline, Arabian Journal of Chemistry 17 (2024) 105912.

[4] K.M. Batoo, K.H. Jassim, T.A. Qassem, S. Hussain, W. Talib Hasson, S.S. Jalal, M.F. Ramadan, S.M. Hameed, A.H. Alawadi, A. Alsaalamy, Novel magnetically separable g-C₃N₄/TiO₂/CuFe₂O₄ photocatalyst for efficient degradation of tetracycline under visible light irradiation: optimization of process by RSM, Journal of Saudi Chemical Society 28 (2024) 101871.

[5] J. Wen, Y. Long, L. Zhu, L. Zeng, X. Qin, S. Song, M. Lu, L. Deng, Bimetallic CuFe₂O₄ photocatalytic activates persistent free radicals and accelerates electron transfer in biochar: efficient degradation of tetracycline, Environmental Research 275 (2025) 121413.

[6] J. Wang, L. Zhang, H. Zhang, R. Cai, H. Jin, M. Xu, X. Cao, S. Yao, Magnetically recyclable spherical Cu₂O@Fe₃O₄ S-scheme heterojunction for efficient tetracycline removal via visible light-activated peroxydisulfate, RSC Advances 14 (2024) 24413–24423.

[7] M. Rajamani, A. Rajan, B. Neppolian, Photocatalytic pathway toward real time control of tetracycline from industrial wastewater mediated by hetero-structure CuFe₂O₄–SnS₂, Journal of Environmental Chemical Engineering 11 (2023) 109129.

[8] S. Ai, S. Zhuo, B. Wang, D.-P. Yang, Photocatalytic activation of peroxodisulfate over the CuFe₂O₄/C/SiO₂ nanocomposite for tetracycline hydrochloride robust degradation, ACS Omega 10 (2025) 24382–24395.

[9] L. Zhu, Y. Zhou, L. Fei, X. Cheng, X. Zhu, L. Deng, X. Ma, Z-scheme CuO/Fe₃O₄/GO heterojunction photocatalyst: enhanced photocatalytic performance for elimination of tetracycline, Chemosphere 309 (2022) 136721.

[10] V.-D. Doan, B.-A. Huynh, H.A.L. Pham, Y. Vasseghian, V.T. Le, Cu₂O/Fe₃O₄/MIL-101(Fe) nanocomposite as a highly efficient and recyclable visible-light-driven catalyst for degradation of ciprofloxacin, Environmental Research 201 (2021) 111593.
